# Establishing a machine learning model based on dual-energy CT enterography to evaluate Crohn’s disease activity

**DOI:** 10.1186/s13244-024-01703-x

**Published:** 2024-05-12

**Authors:** Junlin Li, Gang Xie, Wuli Tang, Lingqin Zhang, Yue Zhang, Lingfeng Zhang, Danni Wang, Kang Li

**Affiliations:** 1 North Sichuan Medical College, Nanchong, 637100 China; 2 Department of Radiology, Chongqing General Hospital, Chongqing, 401121 China; 3 Department of Radiology, Chengdu Third People’s Hospital, Chengdu, 610031 China; 4 Chongqing Medical University, Chongqing, 400016 China

**Keywords:** Inflammatory bowel disease, Crohn’s disease, Dual energy CT, Machine learning, Activity

## Abstract

**Objectives:**

The simplified endoscopic score of Crohn’s disease (SES-CD) is the gold standard for quantitatively evaluating Crohn’s disease (CD) activity but is invasive. This study aimed to develop and validate a machine learning (ML) model based on dual-energy CT enterography (DECTE) to noninvasively evaluate CD activity.

**Methods:**

We evaluated the activity in 202 bowel segments of 46 CD patients according to the SES-CD score and divided the segments randomly into training set and testing set at a ratio of 7:3. Least absolute shrinkage and selection operator (LASSO) was used for feature selection, and three models based on significant parameters were established based on logistic regression. Model performance was evaluated using receiver operating characteristic (ROC), calibration, and clinical decision curves.

**Results:**

There were 110 active and 92 inactive bowel segments. In univariate analysis, the slope of spectral curve in the venous phases (λ_HU_-V) has the best diagnostic performance, with an area under the ROC curve (AUC) of 0.81 and an optimal threshold of 1.975. In the testing set, the AUC of the three models established by the 7 variables to differentiate CD activity was 0.81–0.87 (DeLong test *p* value was 0.071–0.766, *p* > 0.05), and the combined model had the highest AUC of 0.87 (95% confidence interval (CI): 0.779–0.959).

**Conclusions:**

The ML model based the DECTE can feasibly evaluate CD activity, and DECTE parameters provide a quantitative analysis basis for evaluating specific bowel activities in CD patients.

**Critical relevance statement:**

The machine learning model based on dual-energy computed tomography enterography can be used for evaluating Crohn’s disease activity noninvasively and quantitatively.

**Key Points:**

- Dual-energy CT parameters are related to Crohn’s disease activity.
- Three machine learning models effectively evaluated Crohn’s disease activity.
- Combined models based on conventional and dual-energy CT have the best performance.

**Graphical Abstract:**

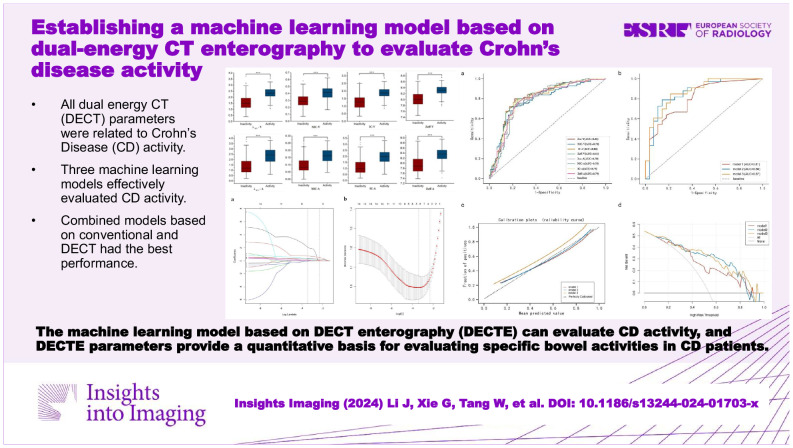

## Introduction

Crohn’s disease (CD) is a chronic, recurrent inflammatory bowel disease (IBD), and its global incidence rate has been increasing, resulting in an extreme economic burden [1, 2]. Progressive inflammation-based stimulation may result in serious complications such as strictures, perforation, or fistulas that require surgery [3]. It is important to monitor the treatment response continuously to adjust medication and guide clinical treatment decisions [4].

The simplified endoscopic score of Crohn’s disease (SES-CD) is the most mature and quantitative scoring system for CD activity; it is limited by its invasiveness, with a risk of serious complications and difficulty in evaluating segments with severe strictures [5, 6]. CT, MR, and intestinal ultrasound have successfully become noninvasive assessment methods for CD due to their ability to visualise the full intestine [7–9]. Some quantitative scoring systems have been developed based on these cross-sectional imaging methods, such as the magnetic resonance activity index, Clermont score, and London score, which mainly reference qualitative parameters [10–12]. However, their clinical practicality has been limited by poor observer consistency and complex calculation methods.

Dual-energy CT enterography (DECTE) is an imaging technique based on two different energy settings for data acquisition that has been widely used in vascular imaging, tumour differentiation, and prognosis in recent years and is now gradually being applied to IBD [13–15]. The Dane team assessed inflammation activity with the iodine concentration (IC) from DECTE and compared it with the results of pathological analysis, indicating that the IC can serve as a radiological marker of CD activity [16]. In addition, some studies have found that the IC, normalised iodine concentration (NIC), and slope of the energy spectrum curve (λ_HU_) are related to CD activity [17–19]. Forty kiloelectron-volt (keV) DECTE contributes to distinguishing wall enhancement between normal and diseased intestinal segments and can improve image quality [20]. Currently, most studies use DECTE to evaluate activity by comparing it with the Crohn’s disease activity index (CDAI) or pathological analysis, with only a few studies comparing it with SES-CD score and finding that NIC and λ_HU_ help differentiate CD activity [21, 22]. This study aimed to analyse and establish an ML model based on DECTE to noninvasively evaluate CD activity with the SES-CD score as the gold standard.

## Materials and methods

### Ethics

This retrospective study was approved by the Ethics Committee of Chongqing General Hospital and exempted from the requirement for written informed consent from patients.

### Patients

**Fig. 1:**
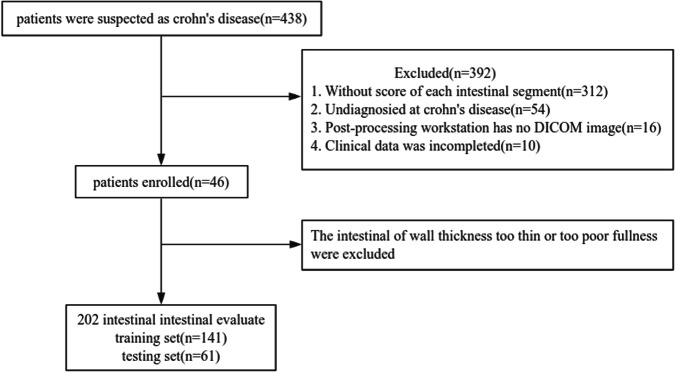
Follow diagram of the study population

Patients suspected as CD at Chongqing General Hospital from July 2021 to 2023 were included, and all subjects underwent DECTE scans. The inclusion criteria for candidate patient selection were as follows: (1) age 18–65 years old, (2) confirmed Crohn’s disease, and (3) endoscopy segmented score obtained within ± 14 days of DECTE scan. The exclusion criteria were as follows: (1) no DECTE image at the postprocessing workstation, (2) poor image quality, such as an intestinal wall that was too thin or had poor fullness, and (3) incomplete clinical data. Clinical data included patient age, sex, course of the disease, disease behavior, drug treatment method (traditional or biological therapy), C-reactive protein (CRP), albumin (ALB), and erythrocyte sedimentation rate (ESR). Finally, 46 CD patients were included, and a total of 202 segments were evaluated. According to the principle of hierarchical randomisation, the data were divided into a training set (*N* = 141) and a testing set (*N* = 61) at a 7:3 ratio. The flow chart of the study population is shown in Fig. 1.

### Endoscopic evaluation

One gastroenterologist who was blinded to the clinical data of each patient performed the endoscopy, divided the whole intestinal segment into five segments (the ileum, right colon, transverse colon, left colon, and rectum), and then assessed inflammation with the SES-CD criteria [23]. Next, they calculated the total score for each bowel, with a score of 0–12 points for each segment. SES-CD < 3 was considered to indicate inactive disease, and SES-CD ≥ 3 was considered to indicate active disease.

### CT scanning

All patients fasted for 8 h before intestinal CTE examination and took 2000 mL 2.5% mannitol solution orally 1 h before examination. All patients were scanned with a dual-energy CT scanner (IQon spectral CT, Philips Healthcare, China). The tube voltage was fixed at 120 kVp, tube current 145 mAs, pitch 1.2, rotation speed 0.5 s, and reconstruction layer thickness 1.00 mm. The contrast agent (Ioversol, 350 mg(I)/mL, Jiangsu Hengrui medicine CO., LTD, China) was administered via a peripheral vein at 1.5 mL/kg and a rate of 3.0 mL/s with a high-pressure syringe. The scanning time was monitored using a monitoring method. The abdominal aorta was detected 10 s after injection of contrast agent, and the threshold was automatically triggered when it reached 150 HU. The arterial and venous phases of DECTE were collected approximately 30 s and 80 s after injection of contrast agent, respectively. We reconstructed dual-energy images at a Philips postprocessing workstation, obtained conventional images at 120 keV, examined decomposed images of water and iodine based on the materials, and obtained monochromatic images within the energy range of 40 to 120 keV.

### Image processing and evaluation

Two radiologists with more than 5 years of expertise in diagnostic abdominal imaging, and who were blinded to the patient information reviewed the traditional CT and DECTE images. The images were evaluated and analysed using the Philips postprocessing workstation. A circular area of interest (ROI) was ideally defined to capture as much of the highly enhanced part of the lesion in the intestinal wall as possible, with a minimum value set at 5 mm^2^ (range: 5 mm^2^–10 mm^2^), and the IC in an artery in the same layer was measured (abdominal aorta or iliac artery). Then, the NIC was calculated as IC in the affected intestinal segment/IC in the artery in the same layer, and the λ _HU_ was calculated as (HU_40 keV_–HU_100 keV_)/60 [24]. The thickness of the intestinal wall, segmental mural hyperenhancement, strictures, upstream dilation, comb sign, fibrofatty proliferation, inflammation, and regional lymph node size were assessed on conventional CT images. To ensure consistency in the results, all measurements were taken three times at different locations on the same layer, and the average value was calculated. Quantitative parameters are ultimately displayed as the arithmetic mean of the value obtained by two radiologists. When qualitative parameters were inconsistent, disagreements were resolved by consensus [25]. The consistency analysis between observers is shown in Supplementary Tables 1 and 2.

### Model construction

The model was constructed with the training set to compare and analyse the clinical, routine imaging, and quantitative parameters of DECTE representing active and inactive intestinal segments. Features with *p* < 0.05 were screened using LASSO regression combined with 10-fold cross validation. We developed three ML models based on conventional image features (model 1), DECTE parameters (model 2), and all significant parameters (model 3) by a logistic regression algorithm and performed parameter tuning by using 5-fold cross validation. Finally, model performance was tested in the testing set.

### Statistical analysis

Python software 3.8 and SPSS 23.0 statistical software were applied to analyse the data. *p* < 0.05 was considered statistically significant. Continuous variables conforming to a normal distribution are expressed as the mean ± standard deviation (SD), and the groups were compared using Student’s *t* test. Continuous variables that did not conform to a normal distribution are presented as medians and interquartile ranges based on their distribution and were compared by the Mann‒Whitney *U* test. Classification data are represented as frequencies (percentages) and were compared using the chi-square test or Fisher’s exact test. Receiver operating characteristic curves, calibration curves and decision curves were used to evaluate model performance.

## Results

### Clinical findings

The demographic and clinical characteristics of the participants are shown in Table 1. This study included 46 CD patients, including 19 males and 27 females, with an average age of 27.50 years [23.00, 33.00]. A total of 202 segments of the intestine were included: 110 segments were active, and 92 segments were inactive. Table 2 shows the distribution of variables in the training and testing sets, indicating that there were no significant differences between the two groups (*p* > 0.05).

**Table 1:** Patient characteristic

Characteristics	Result (*n* = 46)
Gender	
Female	19
Male	27
Mean age (y)	27.500 [23.000, 33.000]
Course of disease (m)	35.000 [21.250, 60.000]
Current medication	
Traditional	5
Biologics	41
Crohn Montreal classification: age at diagnosis	
< 17 y	6
17–40 y	36
> 40 y	4
Crohn Montreal classification: location of disease	
Ileal	2
Colonic	9
Ileocolonic	35
Crohn Montreal classification: behavior	
Nonstricturing, nonpenetrating	20
Stricturing	22
Penetrating and perianal disease	4
Perianal disease	
Yes	24
No	22
C-reactive protein (mg/L)	4.855 [2.180, 16.248]
Erythrocyte sedimentation rate (mm/h)	16.500 [7.750, 28.250]
Albumin (g/L)	43.100 [40.500, 45.400]

**Table 2:** Comparison of baseline characteristic between train set and test set

Parameters	Train (*n* = 141)	Test (*n* = 61)	*p* value
Age (years)	28.000 [23.000, 33.000]	27.000 [21.000, 31.000]	0.116
Sex (F/M)	61/80	25/36	0.764
Wall thickening (mm)	5.000 [3.000, 7.000]	4.000 [3.000, 6.000]	0.299
Segmental mural hyperenhancement (Stratified/Homogeneous)	114/27	48/13	0.723
Strictures (Yes/No)	32/109	12/49	0.204
with upstream dilation (Yes/No)	8/133	7/54	0.149
Fibrofatty proliferation	38/103	20/41	0.400
Engorged vasa recta (Yes/No)	30/101	17/44	0.309
inflammation (Yes/No)	22/119	7/54	0.442
Regional Lymph node (diameter ≥ 0.5) (Yes/No)	57/84	19/42	0.211
Arterial Phase			
Zeff	8.200 [7.780, 8.470]	8.220 [7.860, 8.410]	0.895
IC (mg/mL)	1.580 [0.850, 2.190]	1.670 [0.930, 2.070]	0.759
NIC	0.129 [0.079, 0.180]	0.143 [0.074, 0.197]	0.706
λ _HU_	1.880 [1.070, 2.618]	2.060 [1.088, 2.563]	0.802
Venous Phase			
Zeff	8.240 [8.000, 8.380]	8.150 [7.890, 8.360]	0.194
IC (mg/mL)	1.710 [1.170, 2.010]	1.480 [1.060, 1.970]	0.257
NIC	0.366 ± 0.106	0.335 ± 0.119	0.092
λ _HU_	2.120 [1.465, 2.495]	1.850 [1.323, 2.452]	0.269

*Zeff* Z-effective, *λ*_*HU*_ slope of the energy spectrum curve, *IC* iodine concentration, *NIC* normalised iodine concentration

### Diagnostic performance of DECTE parameters

**Fig. 2:**
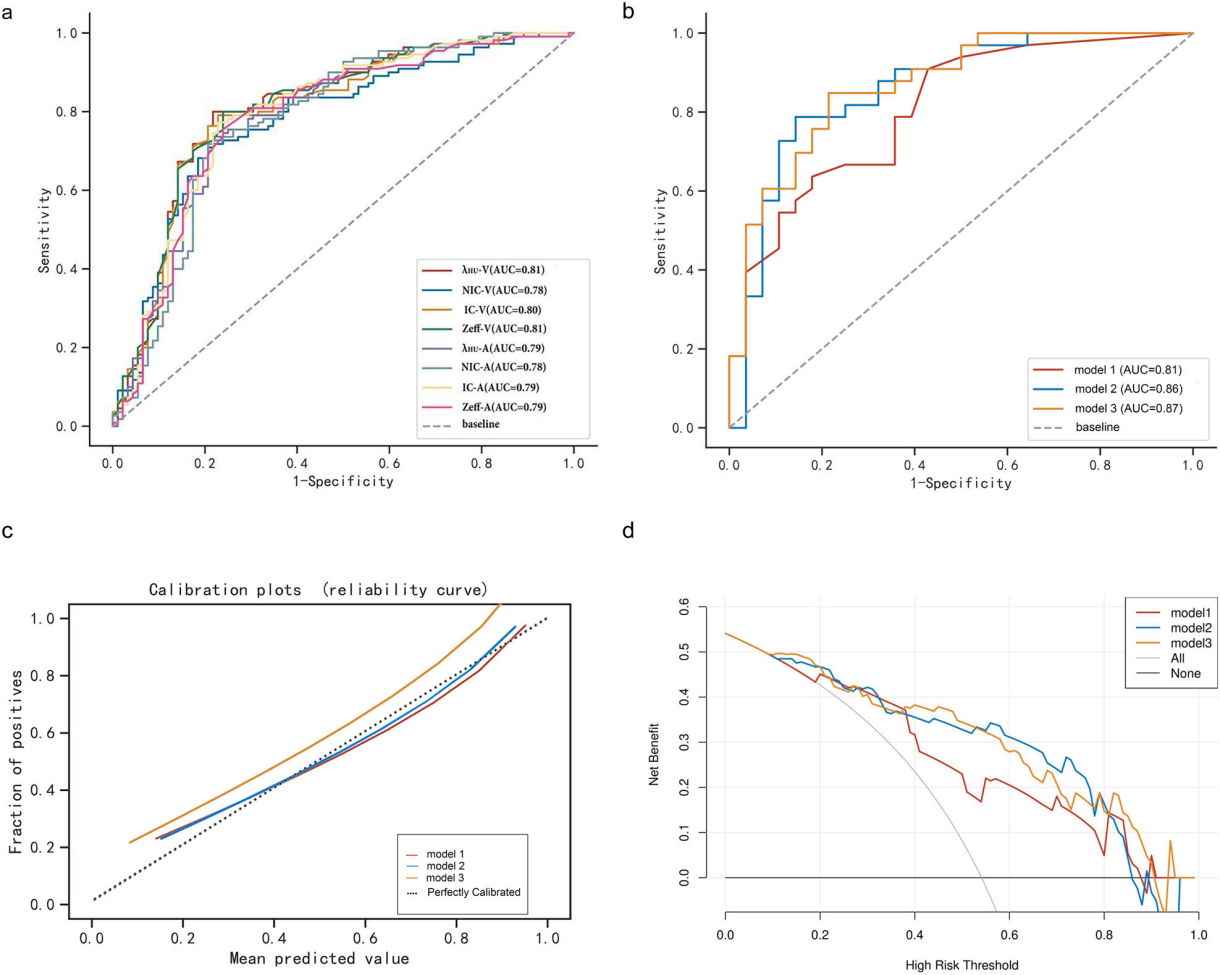
The performance of single DECTE parameters and machine learning models. Receiver operating characteristic curves of single parameters in all sample (**a**). Receiver operating characteristic curves of machine learning models in the test set (**b**). Calibration curves for the three model in testing sets (**c**). Decision curve analysis for the three model in testing sets (**d**)

**Fig. 3:**
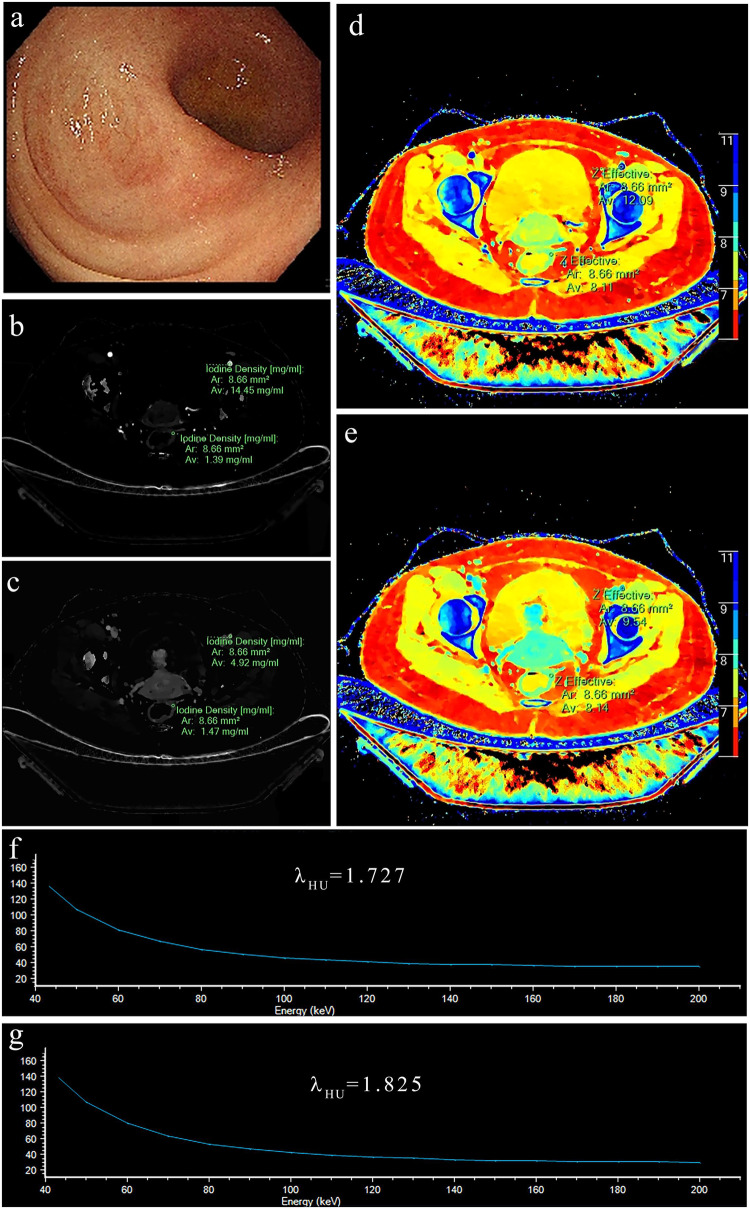
Dual energy CT examination in a 40-years-old female patient with rectum SES-CD score 0. Endoscope, rectum (**a**), iodine centration in the arterial phase (**b**), iodine concentration in the vein phase (**c**), Z-Effective in the arterial phase (**d**), Z-effective in the vein phase (**e**), Slope of the energy spectrum curve in the arterial phase (**f**), Slope of the energy spectrum curve in the vein phase (**g**)

**Fig. 4:**
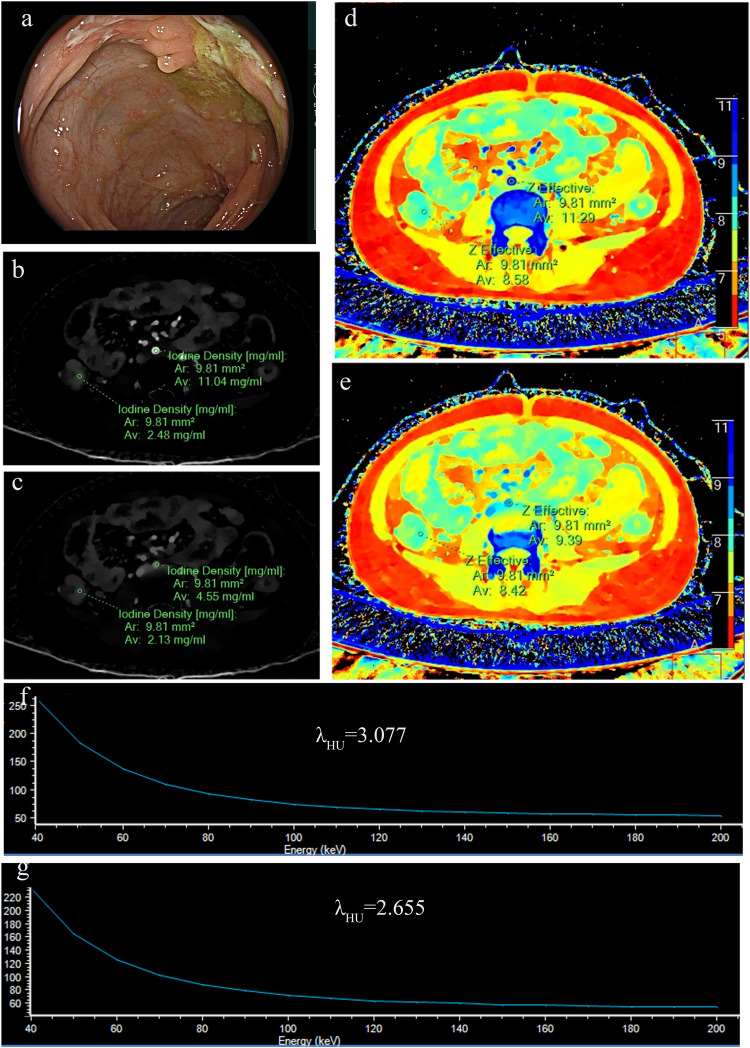
Dual energy CT examination in a 20-years-old female patient with left colon SES-CD score 6. Endoscope, Left colon (**a**), iodine centration in the arterial phase (**b**), iodine concentration in the vein phase (**c**), Z-effective in the arterial phase (**d**), Z-effective in the vein phase (**e**), Slope of the energy spectrum curve in the arterial phase (**f**), Slope of the energy spectrum curve in the vein phase (**g**)

Comparing the DECTE parameters of the active and inactive segments in the total sample, it was found that all parameters of the active intestinal segments were higher than those of the inactive intestinal segments (*p* < 0.001). As demonstrated in Table 3, all DECTE parameters performed well in evaluating CD activity (AUC value > 0.75). λ _HU_ in the venous phase (λ _HU_-V) had the greatest performance in evaluating the activity of CD, with an AUC value of 0.81. When λ _HU_-V ≥ 1.975, its sensitivity and specificity in diagnosing active intestinal segments were 0.800 and 0.783, respectively. According to this result, the ROC curves were plotted in Fig. 2a. Examples of typical images of active and inactive patients are shown in Figs. 3 and 4.

**Table 3:** Evaluation performance of all spectral parameters

Parameters	*N*	AUC (95% CI)	SEN	SPE	YI	Cut-off
Arterial Phase						
Zeff	202	0.785	0.755	0.761	0.515	8.180
IC (mg/mL)	202	0.794	0.773	0.772	0.544	1.550
NIC	202	0.780	0.718	0.793	0.512	0.140
λ _HU_	202	0.787	0.736	0.783	0.519	1.920
Venous Phase						
Zeff	202	0.808	0.800	0.761	0.561	8.190
IC (mg/mL)	202	0.803	0.791	0.772	0.563	1.590
NIC	202	0.777	0.709	0.793	0.503	0.366
λ _HU_	202	0.810	0.800	0.783	0.583	1.975

*Zeff* Z-effective, *IC* iodine concentration, *NIC* normalised iodine concentration, *λ*_*HU*_ slope of the energy spectrum curve, *AUC* area under the curve, *SEN* sensitivity, *SPE* specificity, *YI* Youden’s index, *CI* confidence interval

### Model variable selection

**Fig. 5:**
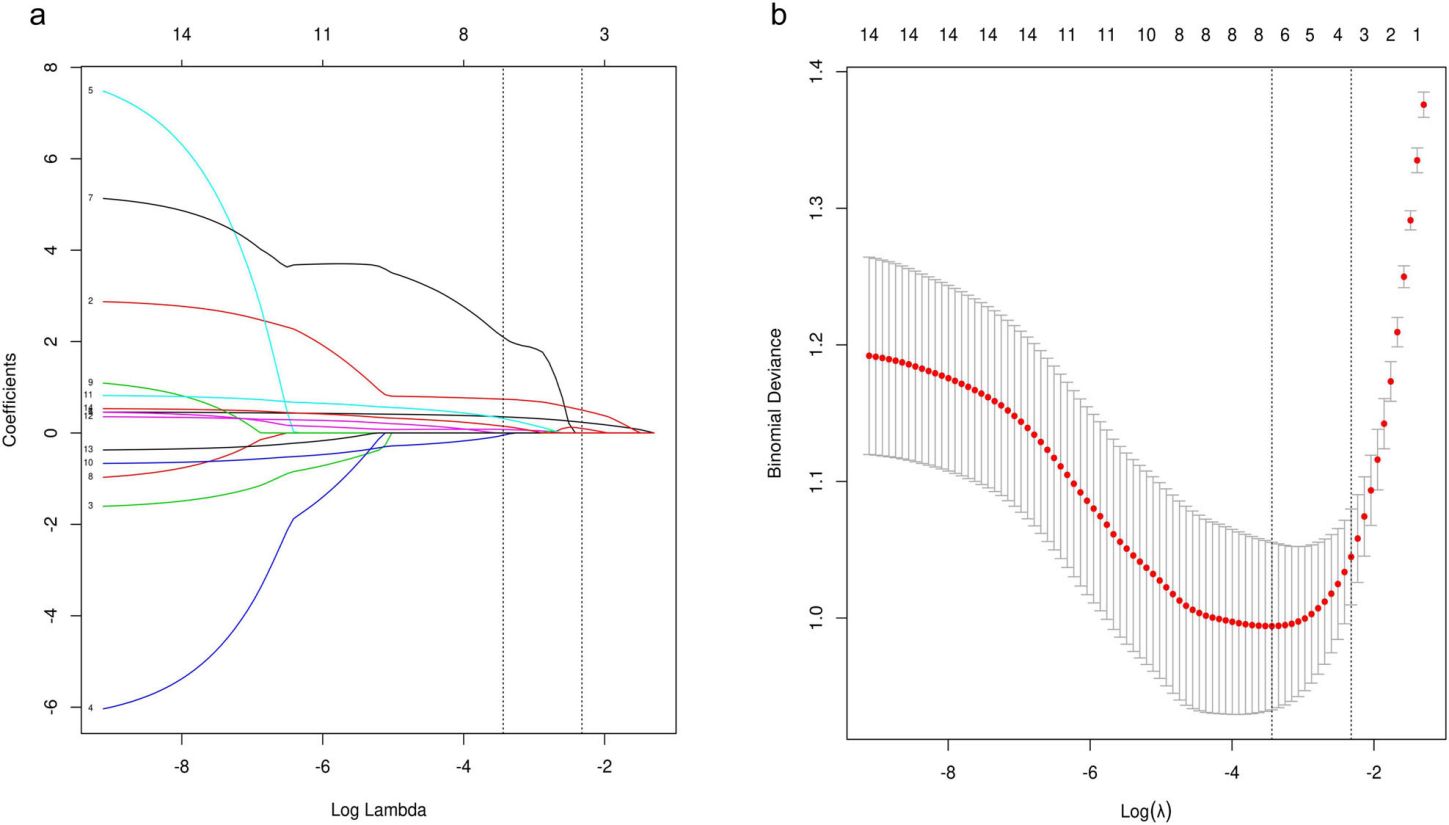
LASSO feature screening pattern diagram. LASSO coefficients for machine learning features (**a**). A coefficient profile plot was generated at the selected log λ value using a tenfold cross-validation, seven machine learning features with the best coefficients were selected. Standard parameters (λ) selection in LASSO model used tenfold cross-validation with a minimum criterion (**b**). The optimal λ values are indicated by the vertical black lines, and a λ value of 0.032 was selected

A comparison between the active and inactive bowels groups in the training set is shown in Table 4, and the results show significant differences (*p* < 0.05) except for age, sex, upstream dilation, and engorged vasa recta. The LASSO algorithm combined with 10-fold cross validation was used to further screen the characteristics when the minimum mean square error (λ was 0.032). Finally, three DECTE parameters and four radiographic features were significant difference: λ _HU_ in the arterial phase, λ _HU_-V, and NIC in the arterial phase, wall thickness, stricture, segmental mural hyperenhancement, and regional lymph node size (Fig. 5).

**Table 4:** Difference between active and inactive segment in training set

Parameters	None-active (*n* = 64)	Active (*n* = 77)	*p* value
Age (years)	28.000 [23.000, 33.000]	29.000 [23.000, 37.000]	0.382
Sex (F/M)	27/37	34/43	0.814
Wall thickening (mm)	3.000 [2.000, 5.000]	7.000 [5.000, 8.000]	< 0.001
Segmental mural hyperenhancement (Stratified/Homogeneous)	6/58	21/56	0.007
Stricture (Yes/No)	3/61	29/48	< 0.001
with upstream dilation (Yes/No)	0/64	8/69	nan
Fibrofatty proliferation	10/54	28/49	0.006
Engorged vasa recta (Yes/No)	0/64	30/47	nan
Inflammation (Yes/No)	3/61	19/58	0.001
Regional Lymph node (diameter ≥ 0.5 cm) (Yes/No)	14/50	43/34	< 0.001
Arterial Phase			
Zeff	7.900 [7.700, 8.160]	8.360 [8.160, 8.535]	< 0.001
IC (mg/mL)	1.030 [0.680, 1.500]	2.010 [1.550, 2.350]	< 0.001
NIC	0.095 [0.065, 0.129]	0.160 [0.120, 0.188]	< 0.001
λ _HU_	1.272 [0.910, 1.775]	2.433 [1.745, 2.923]	< 0.001
Venous Phase			
Zeff	8.076 ± 0.237	8.305 ± 0.191	< 0.001
IC (mg/mL)	1.377 ± 0.481	1.851 ± 0.427	< 0.001
NIC	0.318 ± 0.098	0.405 ± 0.096	< 0.001
λ _HU_	1.691 ± 0.603	2.322 ± 0.515	< 0.001

*Zeff* Z-effective, *IC* iodine concentration, *NIC* normalised iodine concentration, *λ*_*HU*_ slope of the energy spectrum curve

### Diagnostic performance of the machine learning model

The performance indicators of each model in the test set are shown in Table 5, among which model 2 and model 3 had a more balanced overall performance. The three ML models performed well in evaluating CD activity (AUC > 0.80), with the combined model having the highest AUC of 0.87(95% confidence interval (CI): 0.779–0.959) (Fig. 2b). However, the DeLong test showed no statistically significant difference in the AUC among the three models in the test set (the *p* value range of the three models was approximately 0.071 to 0.766, *p* value > 0.05), as detailed in Supplementary Table 3. The calibration curves showed that the fitting curves of the three models almost coincided with the diagonal, indicating a good fit with the actual data (Fig. 2c). Decision curve analysis showed that within the range of approximately 10% to 90%, the clinical net benefits of three models were higher than those of all and none, indicating that all three models had clinical net benefits within a certain threshold probability. Among them, the net benefits of model 2 and model 3 were higher than that of model 1 in the probability range of approximately 38% to 82% (Fig. 2d).

**Table 5:** The performance of three models in testing set

Phase	AUC (95% CI)	Cut off	ACC	SEN	SPE	PPV	NPV	F1
Model 1	0.808	0.526	0.721	0.909	0.571	0.808	0.657	0.855
Model 2	0.858	0.485	0.803	0.788	0.857	0.839	0.767	0.813
Model 3	0.869	0.596	0.77	0.848	0.786	0.852	0.706	0.85

*AUC* area under the curve, *ACC* accuracy, *SEN* sensitivity, *SPE* specificity, *PPV* positive predictive value, *NPV* negative predictive value, *YI* Youden’s index, *CI* confidence interval, *F1* F1 score

## Discussion

This study explored the value of DECTE quantitative parameters in evaluating CD activity and developed ML models for evaluating inflammation in CD patients, including a conventional CT model, a DECTE model, and a combined model. Although there was no significant difference in the AUC among the three models, the DECTE model and the combined model were more balanced in overall performance than the conventional CT model and exhibited better diagnostic performance than individual DECTE quantitative parameters alone.

Among the identified variables, wall thickness and segmental mural hyperenhancement had been previously identified as characteristic parameters in the traditional CT evaluation of CD activity [26, 27]. Literature reports that strictures can also distinguish CD activity [28], and our research confirms these results. Previous studies have shown that lymph node enlargement (length ≥ 1 cm) can be considered a sign of the active stage of CD, but it is more prominent in severely active intestinal segments [27, 29]. We found that there was statistical significance in the size of regional lymph nodes between active and inactive intestinal segments, which could be reactive hyperplasia of mesentery lymph nodes caused by CD activity. This result is contrary to the conclusion of Amir [30], who found that there was no significant difference in the size of regional lymph nodes (diameter > 3 mm) between the active and inactive groups. We believe that this may be firstly due to different reference standards for defining activity–clinical activity scores are nonspecific and cannot represent a certain inflammatory segment or clarify the contribution of the affected segment. Secondly, the included lymph nodes were too small, resulting in statistical insignificance. Although ulceration is an important parameter for CD activity, this study did not evaluate it due to the lower soft tissue resolution of CT compared to MRE.

Zhu et al [21] and Dane et al [16] suggested that NIC is a radiological marker for differentiating active and inactive bowels with SES-CD, which is consistent with the results of our study. We assumed that the outcome may be caused by inflammatory congestion, inflammatory cell infiltration, and noncaseous granulomas in CD patients. λ_HU_ represents the attenuation changes within the lesion during the passage of contrast agent. We found that λ _HU_-V and λ _HU_-A were significantly correlated with CD activity, consistent with previous studies [22, 31], indicating that the amount of contrast agent increases as the blood vessels increase when CD is in the active phase. Our results show that λ _HU_-V had better diagnostic efficacy (AUC 0.81 vs 0.79) than λ _HU_-A, which is consistent with previous research results [31, 32]. This may be because when active inflammation occurs, although the vasa recta expands and increases, the arterial imaging is too early and the contrast agent does not fully enter the lesion. During the venous phase, the contrast agent is fully filled. In addition, when the contrast agent seeps into the extravascular space, the interstitial fibrous tissue can reduce the outflow rate of the contrast agent.

Machine learning is a subset of artificial intelligence. Using feature selection to reduce the dimensions of the data and adjust the hyperparameters can produce a more powerful and generalizable ML model. In recent years, ML in IBD has mainly been used for phenotype diagnosis, gene classification of gut microbiota, and prediction of postoperative recurrence [33–35]. A few studies have constructed ML models to evaluate CD activity and severity. Recently, all ML models constructed by Cai et al [36] performed well in predicting activity in CD test sets. Their study used the CDAI score as the assessment criterion for grouping, while we used the SES-CD as the standard, which displayed the activity of the affected intestinal segment more intuitively compared to CDAI. The Guez [37] team established a multimodal ML model to evaluate CD endoscopic activity by integrating MR information and biochemical indicators. The results showed that the length of diseased intestinal segments and the biochemical indicators were the most informative parameters. In summary, previous research results indicate the potential of ML to accurately and noninvasively assess intestinal activity. Our research also confirms this result. The use of DECTE to establish a ML model provides a new method for non-invasive quantitative evaluation of CD activity, which does not require complex calculations, and the parameters are intuitive and easily acquired. In addition, DECTE scans can reduce scan duration and radiation exposure because of their unique hardware design [38]. The model in this study follows the approach of gastroenterologists in evaluating diseases and specific intestinal segments, revealing the role of different features on the activity of diseased intestinal segments and providing an effective tool for precise clinical diagnosis and treatment decision-making. In addition, compared to traditional statistical methods, machine learning models can usually be rigorously validated.

There are a few limitations that should be noted in this study. First, we used manual ROIs to measure DECTE parameters; in the future, semiautomatic or fully automated methods should be developed to ensure measurement accuracy. Second, our study did not evaluate the correlation between biochemical biomarkers and CD activity, as the relative contribution of each inflammatory segment to the overall biochemical biomarker (such as CRP and ESR, etc.) is unknown. Third, false positive results are a concern in the model. Increasing sample size and using multiple algorithms may be a key factor in reducing false positive rates and improving diagnostic accuracy in the future. Fourth, deep learning is a branch of machine learning and the mainstream trend of future artificial intelligence development. Due to sample size and time constraints, we will further explore the application value of deep learning in Crohn’s disease in the future. Finally, this was a single-centre study whose conclusions require additional validation with multi-centre data before future clinical applications.

## Conclusion

Our machine learning model based on DECTE can feasibly evaluate intestinal segment activity in CD patients, and the DECT parameters provide a quantitative analysis for the evaluation of specific intestinal segment activity in CD patients.

### Supplementary information

Electronic Supplementary Material

## Data Availability

The datasets generated and/or analysed during the current study are not publicly available due to patient privacy regulations but are available from the corresponding author on reasonable request.

## Abbreviations

AUC: Area under the curve
CD: Crohn’s disease
CDAI: Crohn’s disease activity index
CI: Confidence interval
CRP: C-reactive protein
DECTE: Dual-energy CT Enterography
ESR: Erythrocyte sedimentation rate
HU: Hounsfield unit
IBD: Inflammatory bowel disease
IC: Iodine concentration
keV: Kiloelectron volt
LASSO: Least absolute shrinkage and selection operator
NIC: Normalised iodine concentration
ROC: Receiver operating characteristic
ROI: Region of interest
SES-CD: Simplified endoscopic scoring of Crohn’s disease
Zeff: Effective atomic number
λ _HU_: Slope of energy spectrum curve
