# Supplementary material for: Establishing a machine learning model based on dual-energy CT enterography to evaluate Crohn’s disease activity
Source: Insights Imaging. 2024 May 12;15:115. doi: 10.1186/s13244-024-01703-x (PMC11089021; doi:10.1186/s13244-024-01703-x)
Supplement: Supplementary file 1 — Electronic Supplementary Material [file 13244_2024_1703_MOESM1_ESM.pdf]

Establishing a Machine Learning Model Based on Dual-Energy CT Enterography to Evaluate Crohn's Disease Activity

ELECTRONIC SUPPLEMENTARY MATERIAL

Supplementary table 1. Two radiologist consistency in categorical variable

**Parameters**	**kappa**	***p*-value**
Segmental mural hyper enhancement	0.880	<0.001
Stricture	0.794	<0.001
with upstream dilation	0.825	<0.001
Fibrofatty proliferation	0.756	<0.001
Engorged vasa recta	0.800	<0.001
Perienteric inflammation	0.868	<0.001
Lymphadenopathy(0.5cm≤diameter)	0.836	<0.001

Supplementary table 2. Two radiologist consistency in continuous variable

**Parameters**	**ICC**	***p*-value**
Arterial		
Zeff	0.824	<0.001
Iodine concentration(mg/ml)	0.849	<0.001
Normal iodine concentration	0.791	<0.001
λ_{HU}	0.814	<0.001
Portal		
Zeff	0.794	<0.001
Iodine concentration(mg/ml)	0.820	<0.001
Normal iodine concentration	0.776	<0.001
λ_{HU}	0.789	<0.001
Wall thickening (mm)	0.940	<0.001

Zeff Z-effectic, λ_{HU} Slope of the energy spectrum curve

Supplementary table 3. De-long test *p*-value

Name	Model 2	Model 3	Model 1
Model 2		0.766	0.437
Model 3	0.766		0.071
Model 1	0.437	0.071	

Supplementary Table 4. The false positive and false negative tables of the training and testing sets

Name	Training set		Testing set	
	False positive	False negative	False positive	False negative
Model 1	0.160	0.279	0.429	0.091
Model 2	0.273	0.169	0.143	0.212
Model 3	0.180	0.192	0.244	0.152

Supplementary Table 5. Disagreement proportion of qualitative parameters

**Parameters**	***N***	**disagreement**	**Proportion (%)**
Segmental mural hyper enhancement	202	8	0.040
Stricture	202	15	0.074
with upstream dilation	202	5	0.025
Fibrofatty proliferation	202	20	0.099
Engorged vasa recta	202	15	0.074
Perienteric inflammation	202	6	0.030
Lymphadenopathy (0.5 cm≤diameter)	202	6	0.030
